# Estimating the scale of adverse animal welfare consequences of movement restriction and mitigation strategies in a classical swine fever outbreak

**DOI:** 10.1186/s12917-017-1008-5

**Published:** 2017-04-04

**Authors:** Shankar Yadav, Hsin-Yi Weng

**Affiliations:** 1 Department of Comparative Pathobiology, Purdue University, 625 Harrison Street, West Lafayette, IN 47907 USA; 2 Plam Island Animal Disease Center Research Participation Program, West Lafayette, USA

**Keywords:** Classical swine fever, Movement restriction, Pigs, Swine, Outbreak control, Animal welfare, Overcrowding, Risk assessment

## Abstract

**Background:**

The study aim was to quantify the impact of movement restriction on the well-being of pigs and the associated mitigation responses during a classical swine fever (CSF) outbreak. We developed a stochastic risk assessment model and incorporated Indiana swine industry statistics to estimate the timing and number of swine premises that would encounter overcrowding or feed interruption resulting from movement restriction. Our model also quantified the amount of on-farm euthanasia and movement of pigs to slaughter plants required to alleviate those conditions. We simulated various single-site (i.e., an outbreak initiated from one location) and multiple-site (i.e., an outbreak initiated from more than one location) outbreak scenarios in Indiana to estimate outputs.

**Results:**

The study estimated that 14% of the swine premises in Indiana would encounter overcrowding or feed interruption due to movement restriction implemented during a CSF outbreak. The number of premises that would experience animal welfare conditions was about 2.5 fold of the number of infected premises. On-farm euthanasia needed to be performed on 33% of those swine premises to alleviate adverse animal welfare conditions, and more than 90% of on-farm euthanasia had to be carried out within 2 weeks after the implementation of movement restriction. Conversely, movement of pigs to slaughter plants could alleviate 67% of adverse animal welfare conditions due to movement restriction, and only less than 1% of movement of pigs to slaughter plants had to be initiated in the first 2 weeks of movement restrictions. The risk of secondary outbreaks due to movement of pigs from movement restriction areas to slaughter plants was low and only seven pigs from each shipment needed to be tested for CSF infection to prevent a secondary outbreak.

**Conclusions:**

We found that the scale of adverse animal welfare consequences of movement restriction during a CSF outbreak in Indiana was substantial, and controlled movement of pigs to slaughter plants was an efficient and low-risk alternative mitigation response to on-farm euthanasia. The output estimates generated from this study provide empirical evidence for decision makers to properly incorporate required resources for mitigating adverse animal welfare conditions in CSF outbreak management strategic planning.

## Background

After the confirmation of a foreign animal disease (FAD) outbreak in the United States, different countermeasures must be initiated immediately, including designation of control zones, depopulation of infected and contact premises, implementation of movement restrictions, and disease surveillance. Movement restriction is an essential step in containing an outbreak, based on both historical outbreak data [1] and epidemiological simulations [2–5]. According to the FAD outbreak response plan in the United States, no movement of live animals, animal products, vehicles, or people is allowed in a movement restriction area until 28 days after disinfection of the last infected premises [6].

A prolonged period of movement restriction may greatly affect the well-being of pigs. During the 1997–1998 classical swine fever (CSF) outbreaks in the Netherlands, seven million healthy pigs were euthanised due to adverse animal welfare conditions, such as overcrowding and feed interruption, which comprised more than 50% of the total direct costs of the overall outbreak-control initiative [7, 8]. Adverse animal welfare consequences of movement restriction during a CSF outbreak can be particularly problematic in modern intensive pork production systems where pigs are often raised utilising the maximum allowed space of swine premises. Overcrowding may emerge quickly in swine premises if pigs are not moved after reaching their harvest/transition age. Overcrowding may suppress pigs’ biological functioning (e.g., normal growth) and expression of natural behaviours [9, 10]. Obstruction in expression of pigs’ natural behaviours (e.g., exploration or chewing of objects) may trigger tail-biting, ear-chewing, aggression, and fighting [11, 12]. In addition to overcrowding, restricting vehicles into movement restriction areas may interrupt feed supply for swine premises, which can have an immediate impact on the well-being of pigs [13, 14]. Pork producers might also decide to discontinue feed purchases and euthanise the pigs to reduce economic losses during an outbreak.

Despite the significance of this issue, only limited studies have directly assessed animal welfare implications during an FAD outbreak [15, 16]. To address this knowledge gap, our research team initiated a series of studies to quantitatively assess the impacts of movement restriction on the well-being of pigs using CSF as a disease outbreak model [17, 18]. Here we reported the results of a study that integrated the findings from our previous models and Indiana swine industry statistics to estimate the timing and number of swine premises that would encounter adverse animal welfare conditions resulting from movement restriction, and to quantify the frequency of associated mitigation strategies.

## Methods

**Fig. 1:**
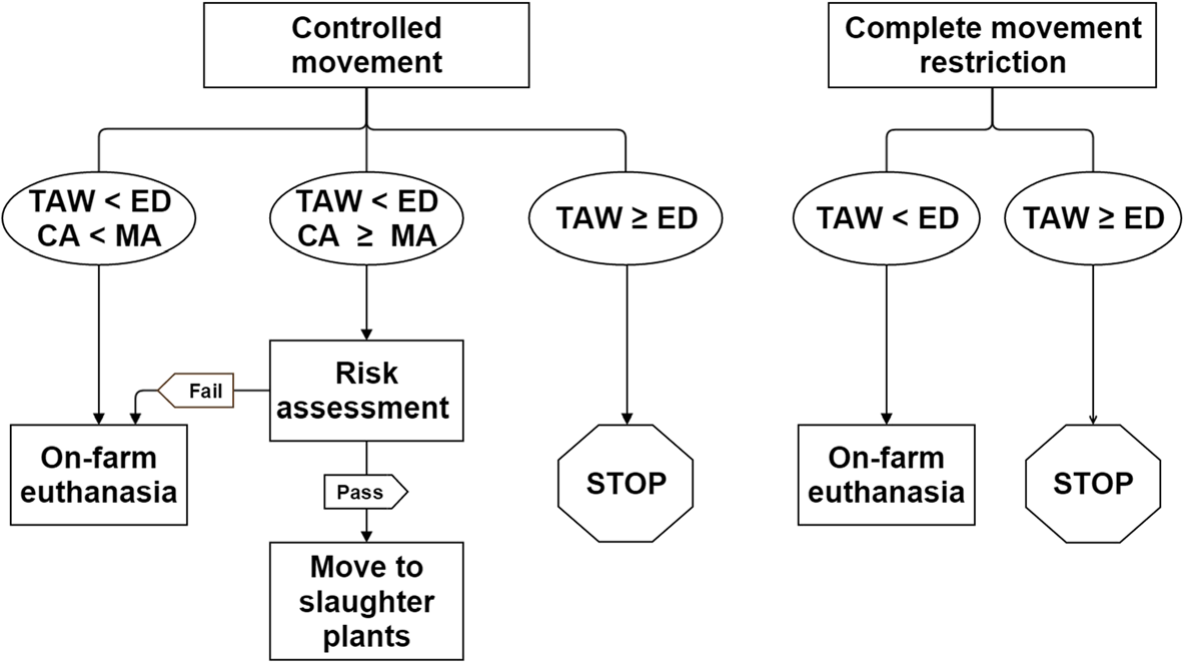
Conceptual framework for the animal welfare mitigation plans under two movement restriction strategies. (*CA*: current age of pigs when animal welfare concerns emerge, *MA*: market-age of pigs, *ED*: epidemic duration, *TAW*: time to adverse animal welfare conditions)

The study started with a roundtable discussion bringing together experts in epidemiology, immunology, animal welfare science, swine veterinary medicine, and pork production to construct a conceptual framework (Fig. 1). During the meeting, we selected overcrowding and feed interruption as the adverse animal welfare consequences of movement restriction for investigation and controlled movement to slaughter plants and on-farm euthanasia as the mitigation strategies for these adverse conditions. We then developed a stochastic risk assessment model based on that conceptual framework and incorporated Indian swine industry statistics to estimate the time elapsed between implementation of movement restriction and onset of overcrowding or feed interruption, the number of swine premises that would encounter adverse animal welfare conditions, and the risk of moving pigs to slaughter plants as a mitigation strategy during a CSF outbreak.

### Conceptual framework

We compiled expert opinions obtained from the roundtable discussion to construct a conceptual framework (Fig. 1) that presented model parameters and decision points for initiating different mitigation responses. Two movement restriction strategies were evaluated: (a) complete movement restriction and (b) controlled movement. Complete movement restriction is the currently recommended CSF control measure in the United States [6], whereas controlled movement is the proposed alternative to be evaluated in this study. Under complete movement restriction, all movement of pigs, people, and vehicles in a designated movement restriction area was prohibited. Under controlled movement, market-age pigs were allowed to be moved to a slaughter plant after a pre-movement risk assessment. The time elapsed between the onset of an outbreak and the emergence of animal welfare conditions such as overcrowding or feed interruption (*TAW*) was compared to epidemic duration (*ED*) to determine the corresponding actions. If *TAW* ≥ *ED*, no overcrowding or feed interruption emerged during an outbreak and no further action was initiated. If *TAW* < *ED*, overcrowding or feed interruption emerged before an outbreak ended and further mitigation responses must be initiated. Under complete movement restriction, on-farm euthanasia was the only option for alleviating these adverse conditions. Under controlled movement, either on-farm euthanasia or movement of pigs to slaughter plants would be initiated to alleviate animal welfare conditions, depending on the current age of the pigs at the time when overcrowding or feed interruption emerged (*CA*). If the pigs had not reached the market age (i.e., *CA < MA*), on-farm euthanasia was initiated; otherwise (i.e., *CA ≥ MA*) the pigs were moved to a slaughter plant after passing the pre-movement risk assessment. If the pre-movement risk assessment identified potential exposure to an infection, on-farm euthanasia was initiated.

### Risk assessment model

We took different steps to develop a novel risk assessment model to estimate the parameters presented in the conceptual framework. In the first step, we established a risk metric to select the most likely CSF outbreak scenarios in Indiana for model simulations in order to estimate *ED* as well as the daily number of newly infected premises during an outbreak. Second, we developed and implemented algorithms to estimate *TAW* and CA. Third, we developed the second set of algorithms to integrate *ED*, *TAW*, and *CA* with Indiana swine industry statistics to estimate the number of swine premises in Indiana that would encounter overcrowding or feed interruption due to movement restrictions during a CSF outbreak and the frequency of different mitigation responses. Fourth, we used the risk assessment model to estimate the number of pigs that needed to be tested from each shipment to prevent a secondary outbreak due to movement to slaughter plants. We modeled nursery (19 to 65 days of age) and finisher (40 to 165 days of age) pork production operations separately. Simulations were run with 100,000 iterations each unless otherwise stated.

### Simulation of the most likely CSF outbreak scenarios to estimate ED

We developed a risk metric for selecting the most likely CSF outbreak scenarios in Indiana using different data sources, including the 2012 Indiana State Swine Premise Identification Database (USAHERDS), United States Census data, and 2013 Indiana State Natural Resources data. Based on the risk metric, we selected 19 single-site (i.e., outbreaks initiated from one location) and 15 multiple-site (i.e., outbreaks initiated from more than one location) CSF outbreak scenarios for the simulations to estimate probability distributions of *ED*. We performed the simulations using the North American Animal Disease Spread Model (NAADSM), a stochastic, temporal, and spatial state transition disease spread model [19]. Details on the development of the risk metric, the selection of outbreak scenarios, and the estimations of *ED* can be found in a previous study [17].

The simulation results of single-site outbreaks revealed a bimodal distribution of *ED* [17]. Therefore, we assigned two triangular distributions [denoted as Triangle (minimum, most likely, maximum)], Triangle (24, 50, 100) and Triangle (100, 224, 343), to present the two clusters of *ED* for a single-site CSF outbreak. In addition, we assigned a Bernoulli distribution [denoted as Bernoulli (probability of event)], Bernoulli (0.3) for Triangle (24, 50, 100). We randomly selected four multiple-site outbreak scenarios from the 15 scenarios to estimate the study outputs. Table 1 presents different triangular distributions for the *ED* for the sampled multiple-site outbreaks.

**Table 1:** Probability distributions for epidemic duration in different classical swine fever outbreak scenarios in Indiana, USA

Outbreak scenario	Probability distribution
Single-site outbreak^a^	Cluster 1: Triangle (24, 50, 100) Cluster 2: Triangle (100, 224, 343) Probability of cluster 1: Bernoulli (0.3)
Multiple-site outbreak with 4 index premises^b^	Triangle (29, 203, 514)
Multiple-site outbreak with 17 index premises^b^	Triangle (138, 185, 341)
Multiple-site outbreak with 20 index premises^b^	Triangle (142, 187, 440)
Multiple-site outbreak with 26 index premises^b^	Triangle (146, 197, 311)

The results were generated from the simulation with 500 iterations using the North American Animal Disease Spread Model

^a^The results are for four single-site outbreak scenarios combined

^b^Index premises are infected premises when an outbreak starts

### Risk assessment model to estimate TAW and CA

In this study, we defined overcrowding as a condition in which the total weight of pigs on premises exceeds 100–115% of the maximum capacity of that premises. The maximum capacity of premises was quantified by the total weight of pigs at the harvest/transition age on that premises. We implemented algorithms to compute the daily total weight of pigs on premises and compared it to the threshold weight that defined overcrowding. The model implemented those algorithms to flag the time at which the total weight exceeded the threshold (i.e., when overcrowding occurred).

We modeled feed interruption to emerge shortly after a swine producer decided to discontinue feed supply and euthanise the pigs on premises in order to reduce economic losses due to a CSF outbreak. We implemented three decision attributes in the algorithms that would increase the probability for a producer to discontinue feed supply: (a) when the estimated epidemic duration was longer than a producer’s tolerance level; (b) when the time interval between the age of pigs at the onset of the outbreak and the harvest/transition age was longer than a producer’s tolerance level; and (c) when the progression of an ongoing outbreak lasted longer than a producer’s tolerance level. The tolerance level of a producer was modeled by the uniform distributions between 14 and 46 days and between 30 and 125 days for nursery and finisher operations, respectively. For simplicity, we assigned the probability for a producer to discontinue feed supply to high [Bernoulli (0.3)] or low [Bernoulli (0.05)] depending these three decision attributes. High probability was implemented when any of the three decision attributes exceeded a producer’s tolerance level; otherwise low probability was implemented.

We quantified *TAW* as the number of days between the implementation of movement restriction and the onset of overcrowding or feed interruption, whichever occurred first. We then computed *CA* as the age of pigs at the onset of an outbreak plus *TAW.* In the model we assumed that (a) movement restriction would be implemented within 24 h of the detection of an outbreak and (b) the age of pigs represented the oldest age among pigs on the same premises. With the latter assumption, the simulations returned the shortest *TAW* for that premises. Based on the results, we assigned Triangle (2, 14, 59) and Triangle (2, 17, 59) for *TAW* in nursery operations in single-site outbreaks and multiple-site outbreaks, respectively [18]. Triangle (2, 14, 260) and Triangle (2, 14, 253) were assigned for *TAW* in finisher operations in single-site outbreaks and multiple-site outbreaks, respectively [18]. Details of the model algorithms for estimating *TAW* are described in a previous study [18].

### Estimation of the scale of adverse animal welfare conditions and mitigation responses

In this final step of model building, we implemented 2012 Indiana swine industry statistics compiled in USAHERDS in the risk assessment model, incorporating the input parameters generated in the previous steps. We did so to estimate the number of swine premises in Indiana that would encounter overcrowding or feed interruption during a CSF outbreak, the scope of corresponding mitigation responses, and the risk of secondary outbreaks due to movements of pigs from movement restriction areas to slaughter plants. The data compiled in USAHERDS included identification number, herd size, operation type, and geolocation of premises.

Based on the United States CSF response plan, a movement restriction area was designated as a circular area 7 km away from the perimeter of an infected zone. We identified the locations of infected premises at the onset of an outbreak for a sample of four single-site and six multiple-site most likely CSF outbreak scenarios. Movement restriction areas were mapped for each infected premises and the number of unique swine premises in movement restriction areas was determined. We used those results to derive a logarithmic regression equation (Table 2) to estimate the number of unique swine premises that fell under movement restrictions given the number of infected premises. In addition to the four multiple-site outbreak scenarios described previously (Table 1), two scenarios with the number of index premises of 23 and 32 were included in the computation of regression equation. The regression line had an R^2^ = 0.945, indicating a good fit to the data.

**Table 2:** Parameters for estimating the number of premises encountering animal-welfare conditions during a CSF outbreak

Parameter	Description	Distribution and algorithm
Number of daily new infections (*N_inf*)	This parameter was derived from the simulations of four single-site and four multiple-site CSF outbreak scenarios in Indiana (500 iteractions each). The mean and standard error (SE) of number of daily new infections were computed. Normal (0,1) represnted the distribution for number of SE. Thus, the estimates of *N_inf* varied around an overall mean by different numbers of SE.	*N_inf*_*i*_ = Mean_*i*_ + Normal (0,1) × SE_*i*_ on day *i*
Number of premises under movment restriction (*N_MR*)	This parameter was estimated as a function of the number of infected premises. Logarithmic regression equation was derived from the simulations of four single-site and six multiple-site CSF outbreak scenarios in Indiana. Normal (17,36) was dedrived from the regression residuals.	*N_MR*_*i*_ = 201.5 × LN $\left({\sum}_{t=0}^{t= i} N\_{\mathit{\inf}}_t\right)$+ Normal (17,36) on day *i*
Index for *TAW* < *ED* (*Index*)	The probability distributions for *TAW* and *ED* were published in previous studies [17, 18]. A binary index was created to flag *TAW* < *ED* (i.e., *Index* = 1; else *Index* = 0), indicating that animal-welfare conditions occurred before an outbreak ended.	Number of premises that encountered animal-welfare consequencs of movement restriction on day *i* = *N_MR*_*i*_ × *Index*_*i*_

*TAW* The time elapsed between the onset of an outbreak and the emergence of animal welfare conditions, *ED* Epidemic duration

We simulated the selected eight CSF outbreak scenarios using NAADSM to estimate the number of daily new infections. Five hundred iterations were simulated for each outbreak scenario, and the average daily number of new infections and standard error were computed for all single-site outbreak scenarios combined and for individual multiple-site outbreak scenarios. We implemented the estimates of number of new infections in the regression equation to estimate the number of unique swine premises that fell under movement restrictions. We also modeled standard errors for the number of new infections and regression residuals to account for variations. The model algorithms then computed *TAW* for premises under movement restrictions on a particular day and compared it to *ED* to identify premises that would encounter overcrowding or feed interruption before an outbreak ended (i.e., *TAW* < *ED*). Among them, the model compared *CA* to *MA* to determine whether on-farm euthanasia (i.e., *CA* < *MA*) or movement of pigs to slaughter plants (i.e., *CA* ≥ *MA*) should be initiated to alleviate the adverse animal welfare conditions on that premises (Fig. 1). Using the risk assessment model, we estimated the total number of swine premises that would encounter overcrowding or feed interruption and the amount of on-farm euthanasia and movement of pigs to slaughter plants to be initiated during a CSF outbreak in Indiana. Table 2 presents details on model input parameters and algorithms for this step of risk assessment.

The final step of model building was to estimate the maximum threshold risk that would not trigger a secondary outbreak due to movement of pigs from movement restriction areas to slaughter plants. We then used that maximum threshold risk to estimate the number of pigs that had to be sampled and tested for CSF from each shipment to detect an infection. To accomplish this objective, we included all listed slaughter plants in USAHERDS as potential receipting sites in the disease spread simulations using NAADSM. To determine the maximum threshold risk, we simulated various levels of within-premises CSF prevalence of subclinical infections to manipulate the disease transmission rate of direct contact in NAADSM. The model assumed that pigs were held at a slaughter plant for no more than 24 h before being processed. With this assumption and the assumption that pigs showing clinical signs of CSF would be inevitably identified, only pigs that were at the subclinical stage of CSF infection on premises before movement had the chance to spread the disease while at a slaughter plant. This was because the average latent and subclinical periods of CSF were 4 days and 6 days, respectively [20]. In NAADSM, infected pigs were assumed to start shedding the virus at the subclinical stage of CSF. The rate of disease transmission through indirect contact was adopted from a previous study [17].

### Sensitivity analyses

We performed sensitivity analyses to identify influential input parameters. In the first step, we used Spearman’s correlation to identify parameters that were associated with the target output (e.g., number of premises that would encounter adverse animal welfare conditions). We further investigated the parameters that showed a correlation coefficient ≥ 0.3 using simulations of different input values to quantify their influences on output estimates.

## Results

There were 8631 swine premises listed in Indiana USAHERDS in 2012; 86% of them were small operations (herd size range: 1–699) and 14% were commercial (herd size range: 200–20,000). The estimated *TAW* was similar between the single-site and multiple-site outbreak scenarios, with a median (25th, 75th percentiles) of 19 days (10, 29) in nursery operations and 57 days (13, 93) in finisher operations [18].

**Fig. 2:**
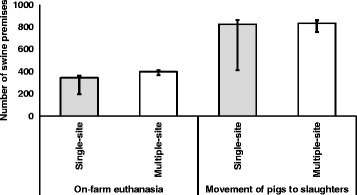
Number of premises that required mitigation of animal welfare conditions during a CSF outbreak in Indiana. (The *bar chart* represents the median and *error bars* at the 25th and 75th percentiles. The results were generated from simulations of four single-site (i.e., an outbreak initiated from one location) and four multiple-site (i.e., an outbreak initiated from more than one location) outbreak scenarios in Indiana)

**Fig. 3:**
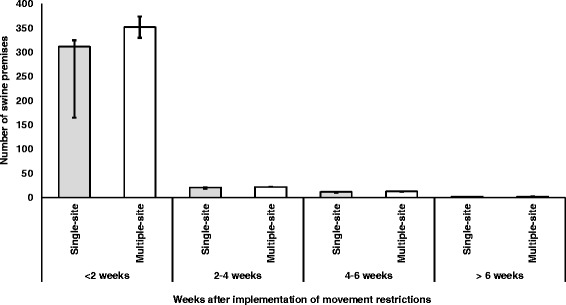
Timing of on-farm euthanasia to alleviate animal welfare conditions during a CSF outbreak in Indiana. (The *bar chart* represents the median and *error bars* at the 25th and 75th percentiles. The results were generated from simulations of four single-site (i.e., an outbreak initiated from one location) and four multiple-site (i.e., an outbreak initiated from more than one location) outbreak scenarios in Indiana)

**Fig. 4:**
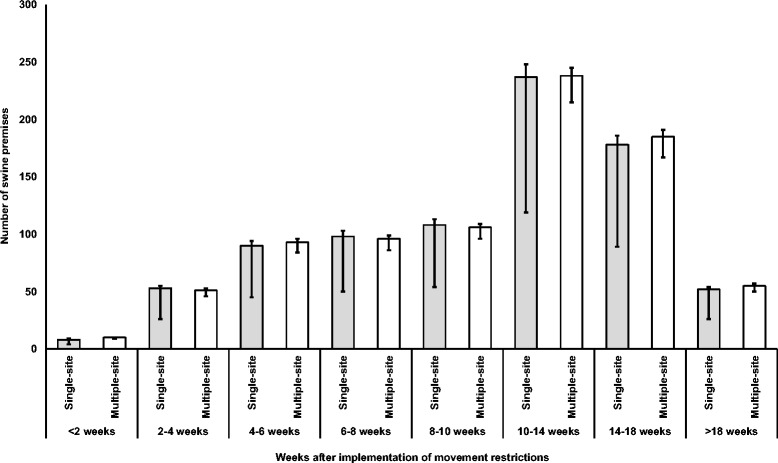
Timing of movement to slaughter as an animal welfare mitigation during a CSF outbreak in Indiana. (The *bar chart* represents the median and *error bars* at the 25th and 75th percentiles. The results were generated from simulations of four single-site (i.e., an outbreak initiated from one location) and four multiple-site (i.e., an outbreak initiated from more than one location) outbreak scenarios in Indiana)

The median (25th and 75th percentiles) numbers of infected swine premises and swine premises that would encounter animal welfare consequences of movement restriction for all four single-site CSF outbreak scenarios combined were 466 (443, 488) and 1169 (603, 1224), respectively (Table 3). The median numbers of infected swine premises ranged from 468 to 614 in the four multiple-site outbreak scenarios, and the median numbers of swine premises that would encounter animal welfare consequences of movement restriction ranged from 1170 to 1293 (Table 3). Among them, approximately 5% were nursery and 95% were finisher operations. On-farm euthanasia would need to be initiated for about 33% of the swine premises that encountered adverse animal welfare conditions, whereas movement of pigs to slaughter plants could be initiated for about 67% of the swine premises to alleviate adverse animal welfare conditions (Fig. 2). On-farm euthanasia was carried out for all nursery operations and for approximately 28% of finisher operations that encountered adverse animal welfare conditions, and about 90% of the on-farm euthanasia was initiated within the first 2 weeks of the implementation of movement restriction (Fig. 3). In comparison, only 1% of movement of pigs to slaughter plants was initiated within the first 2 weeks of movement restriction (Fig. 4).

**Table 3:** Numbers of infected swine premises and premises with animal welfare conditions during a CSF outbreak

Outbreak scenario	Infected premises	Premises with animal welfare conditions
Single-site outbreak^a^	466 (443, 488)	1169 (603, 1224)
Multiple-site outbreak with 4 index premises^b^	467 (446, 488)	1182 (1016, 1231)
Multiple-site outbreak with 17 index premises^b^	590 (575, 606)	1277 (1233, 1307)
Multiple-site outbreak with 20 index premises^b^	609 (596, 621)	1293 (1259, 1321)
Multiple-site outbreak with 26 index premises^b^	614 (602, 625)	1289 (1248, 1319)

Numbers are the median (25th and 75th percentiles)

^a^The results are for four single-site (i.e., an outbreak starts with one infected premises) CSF outbreak scenarios in Indiana combined

^b^Index premises are infected premises when an outbreak starts

Using the risk assessment model, we found that the risk of secondary outbreaks due to movement of pigs from movement restriction areas to slaughter plants was very low. Such movements would not trigger a secondary outbreak even with the within-premises prevalence of CSF as high as 50%. We further estimated that each swine premises was allowed to have a maximum of five movements during an outbreak, and seven pigs should be sampled and tested for CSF before each shipment to detect an infection with a confidence level of 99%.

In the initial sensitivity analyses, we identified that *TAW* for finisher operations was negatively, and *ED* positively, correlated with the estimate of number of premises experiencing adverse animal welfare consequences of movement restriction. In further investigations, a ± 10% margin of the original input probability distributions of those two input parameters resulted in a less than 1% change in the output estimates.

## Discussion

The aim of this study was to estimate the scale of adverse animal welfare consequences of movement restriction and associated mitigation responses during a foreign animal disease outbreak that affects swine. We simulated different CSF outbreak scenarios combining with Indiana swine industry data to estimate outputs. Using the risk assessment model, we estimated that approximately 14% of the swine premises in Indiana would encounter adverse animal welfare conditions that resulted from movement restrictions imposed as one of the countermeasures for outbreak control. According to the 2012 U.S. Census of Agriculture, Indiana produced about 11 million pigs in 2012 [21], and, therefore, 1.5 million pigs would need to be euthanised on-farm or moved to slaughter plants as a mitigation of adverse animal welfare conditions due to movement restrictions during a CSF outbreak. The estimates varied depending on the characteristics of an outbreak, with a multiple-site outbreak resulting in slightly more swine premises with animal welfare consequences compared to a single-site outbreak. However, the differences in the estimates of the total number of both infected premises and premises with animal welfare consequences between the two types of outbreak were negligible (Table 3). The number of premises that would encounter animal welfare conditions was approximately 2.5 fold of the number of infected premises. These estimates provide valuable information for decision makers to develop proper strategic plans for alleviating animal welfare consequences of movement restrictions during a CSF outbreak. Although we used the Indiana swine industry data to simulate the estimates, the finding that the scale of animal welfare consequences of movement restrictions was substantial and greater than the number of infections agreed with findings based on historical CSF outbreaks in Europe [1, 22, 23]. Our risk assessment model could be easily adapted by other states in the United States that have a similar pork production system to generate state-specific estimates.

The timing of initiating on-farm euthanasia or movement of pigs to slaughter plants to alleviate animal welfare conditions is also a critical parameter for planning CSF outbreak management. Our model showed that about 90% of on-farm euthanasia had to be initiated within 2 weeks of movement restrictions. This finding suggested a competing resource for depopulating infected and contact premises in an infected zone that was required to be completed as soon as possible after the identification of an infection. It could greatly hinder outbreak control efforts if the high demand of animal welfare alleviation response activities was not properly considered in outbreak response plans. For example, during the 2001 ft-and-mouth disease outbreaks in the United Kingdom, slaughter staff, labor, and carcass disposal sites were found to be insufficient mainly due to inadequate consideration of animal welfare implications [24]. In the 1997–1998 CSF outbreaks in the Netherlands, the number and capacity of rendering plants were insufficient to dispose of the large volume of swine carcasses that were mostly from the euthanasia of pigs for alleviating adverse-welfare conditions [22, 25, 26]. In contrast to on-farm euthanasia, our model showed that only less than 1% of movement of pigs to slaughter plants would need to be initiated within 2 weeks of movement restrictions. This mitigation strategy could greatly reduce pressure from competing limited resources for outbreak controls.

Movement of pigs to slaughter plants may also help moderate food insecurity concerns due to discontinuation of the pork supply chain [27, 28]. Consequently, it can significantly reduce the economic losses of pork producers. Furthermore, euthanasia of a large number of animals, particularly healthy animals, may affect livestock owners, their families, and people involved in executing mass depopulation not only economically but also psychologically [29–31]. Mass euthanasia could pose negative public and social perception of the agriculture industry, detrimental effects on the local tourist industry, and delayed recovery and resumption of normal reproduction of livestock. In their study, Thompson et al. [30] showed that inappropriate carcass disposal of euthanised animals during the 2001 FMD outbreak in UK may have contributed substantially to an estimated $3.2 billion in losses to the country’s tourist industry. We demonstrated that movement of pigs to slaughter plants can greatly reduce the amount of on-farm euthanasia for mitigating adverse animal welfare conditions during a CSF outbreak. Based on the study model, movement of pigs to slaughter plants could be initiated to alleviate approximately 67% of pigs that experienced adverse-welfare conditions in Indiana.

During a CSF outbreak, all swine premises that are under movement restrictions are under surveillance for potential infection. A detection of infections will prompt the designation of new control zones surrounding infected premises. However, even with ongoing surveillance it is still vital to conduct pre-movement risk assessment to prevent a subclinical pig from carrying disease outside a control zone. Our model showed that a limited number of controlled movements (i.e., ≤5 per premises) of pigs from movement restriction areas to slaughter plants posed very low risk for secondary outbreaks. With such a low risk, only seven pigs have to be sampled and tested for CSF infection from each shipment to detect an infection with a 99% confidence level. This amount of pre-movement risk assessment is considered practicable during a CSF outbreak, owing to the advances in CSF diagnostic tests. The World Organisation of Animal Health (OIE) recommends different diagnostic techniques for CSF infections or exposures in pigs, such as conventional reverse transcription polymerase chain reaction (RT-PCR), antigen enzyme linked immune-sorbent assay (ELISA), fluorescence antibody test (FAT), and virus neutralization [32]. Among them, RT-PCR is considered the most sensitive and reliable test for quick CSF diagnosis during an outbreak [33–35]. Studies have shown that infected pigs could be detected as early as 2 days post-infection and 3 to 4 days before onset of clinical signs using RT-PCR [36, 37]. In addition, RT-PCR could generate test results in 2 h [37]. Therefore, we recommend applying RT-PCR in pre-movement risk assessment for the mitigation strategy of movement of pigs to slaughter plants during a CSF outbreak.

## Conclusions

The scale of animal welfare consequences of movement restrictions during a CSF outbreak in Indiana was substantial. Movement of pigs to slaughter plants was an efficient and low-risk alternative to on-farm euthanasia to alleviate adverse animal welfare conditions. Our risk assessment model estimated that movement of pigs to slaughter plants could be used to alleviate approximately 67% of the animal welfare consequences of movement restrictions in Indiana. Seven pigs needed to be tested for CSF infection in the pre-movement risk assessment to ensure the prevention of a secondary outbreak.

## Abbreviations

CA: Current age of pigs at the onset of animal welfare conditions
CSF: Classical swine fever
ED: Epidemic duration
FAD: Foreign animal disease
MA: Market-age of pigs
NAADSM: North American Animal Disease Spread Model
TAW: The time elapsed between the onset of an outbreak and the emergence of animal welfare conditions
